# Characteristics of recurrence in area postrema-onset NMO spectrum disorder - a retrospective cohort study

**DOI:** 10.1186/s12883-024-03667-3

**Published:** 2024-05-21

**Authors:** Xianxing Zhang, Jin Wu, Jingyu Lin, Shifang Lin, Aiyu Lin

**Affiliations:** 1 Department of Neurology, the First Affiliated Hospital of Fujian Medical University, Fuzhou, Fujian 350005 China; 2 Fujian Key Laboratory of Molecular Neurology, Institute of Neuroscience, Fujian Medical University, Fuzhou, Fujian 350004 China; 3 Department of Neurology, National Regional Medical Center, Binhai Campus of the First Affiliated Hospital of Fujian Medical University, Fuzhou, Fujian 350212 China; 4 Department of Inspection four, Fujian Center for Drug Inspection and Fujian Center for Vaccine Inspection, Fuzhou, Fujian 350004 China

**Keywords:** Area Postrema, Neuromyelitis Optica spectrum disorder, Annual recurrence rate

## Abstract

**Background:**

Neuromyelitis Optica Spectrum Disorder (NMOSD) is an inflammatory autoimmune disease with high risk of recurrence and disability, the treatment goal is a recurrence free state. Area postrema (AP) is one of the most common involved area of NMOSD, which may have a particular significance in the pathogenesis of NMOSD and clinical heterogeneity. Our study is to investigate the clinical and recurrent characteristics AP onset NMOSD patients.

**Methods:**

A retrospective study was done in a cohort of 166 AQP4-IgG seropositive NMOSD patients which were identified by the 2015 IPND criteria. The patients were divided into AP onset (APO-NMOSD) group and non-AP onset (NAPO-NMOSD) group based on the initial episode location. Clinical features and recurrence differences of two groups were compared.

**Results:**

The APO-NMOSD group and NAPO-NMOSD group had a population ratio of 24:142. APO-NMOSD patients were younger (34.6y VS 42.3y, *P* = 0.013), had lower EDSS at first episode (0.7 VS 4.2, *p* = 0.028) and last follow up (1.9 VS 3.3, *p* = 0.001), more likely to have multi-core lesions at the first attack (33.3% VS 9.2%, *P* = 0.001). Also, they had a higher annual recurrence rate (0.4 ± 0.28 VS 0.19 ± 0.25, *P* = 0.012). In natural course NMOSD patients without immunotherapy, APO-NMSOD had a shorter time of first relapse (*P* < 0.001) and higher annual recurrence rate (0.31 ± 0.22 VS 0.16 ± 0.26, *P* = 0.038) than NAPO-NMOSD. APO-NMOSD group also have a higher risk of having the first relapsing compared to optic neuritis onset-NMOSD (HR 2.641, 95% CI 1.427–4.887, *p* = 0.002) and myelitis onset-NMOSD group (HR 3.593, 95% CI 1.736–7.438, *p* = 0.001). Compared to NAPO-NMOSD, APO-NMOSD has a higher likelihood of brainstem recurrence (28.6% vs. 4.7%, *p*<0.001) during the first recurrence, while NAPO-NMOSD is more susceptible to optic nerve involvement (10.7% vs. 41.1%, *p* = 0.01).

**Conclusion:**

AQP4-IgG seropositive NMOSD patients with AP onset are youngers and have higher risk of recurrence. Clinicians should pay attention to AP damage in NMOSD, as it indicates a potential risk of recurrence.

**Trial registration:**

Retrospectively registered.

## Background

Neuromyelitis optica spectrum disorder (NMOSD) is an inflammatory autoimmune disease primarily mediated by Aquaporin-4 (AQP4) antibodies [1], that preferentially affects AQP4-riched regions such as the optic nerve, spinal cord, area postrema (AP), brainstem and diencephalon. Area Postrema Syndrome (APS) usually begins with unexplained episodes of intractable hiccups and/or nausea and vomiting. Mild disability and the lack of specificity of symptoms often lead to neglect, misdiagnosis and delayed treatment [2–4]. AP is thought to be the “entrance” of AQP4-IgG causing immune attack in NMOSD because of the high expression of AQP4 and the transmission of substances from the blood into the cerebrospinal fluid due to the capillaries lacks intercellular tight junction [5–8]. It is not known whether AP has a particular significance in the pathogenesis of NMOSD and clinical heterogeneity. Therefore, it may be of great clinical significance to investigate the characteristics and recurrence of AQP4-IgG seropositive NMOSD with the AP onset.

## Methods

### Case selection and data collection

Clinical information and samples for this observational, retrospective study were collected from The First Affiliated Hospital of Fujian Medical University from September 2017 to June 2022. This study was conducted as part of a Registered Cohort Study of Inflammatory Demyelination Disease (NCT04386018). A total of 166 patients diagnosed with NMOSD according to the 2015 IPND criteria [9]. Epidemiologic data, including demographic, clinical and MRI findings, treatment, and outcome were obtained from medical records and information collected through a structured questionnaire designed for NMOSD as reported [10]. All the patients were divided into AP-onset NMOSD group and non-AP-onset NMOSD group based on the initial lesion. The clinical characteristics and recurrence of APO-NMOSD patients were compared with that of NAPO-NMOSD.

The serum samples were tested for AQP4-IgG by an in-house cell-based assay with live HEK293 cells transfected with the aquaporin-4-M23 isoform [11, 12]. An attack was defined as the presence of a new symptom(s) or worsening of an existing symptom(s) lasting at least 24 h and accompanied by new neurologic findings, occurring 30 days after the previous attack. The first relapsing time and lesion of all the patients were recorded. The last follow-up visit was evaluated by the Expanded Disability Status Scale (EDSS) score [13]. An EDSS score of 6.0 was attributed when the patient required intermittent or unilateral assistance to walk 100 m with or without resting. Severe visual disability was defined as sustained visual acuity (VA) ≤ 10/100 with best correction possible during at least 6 months after an optic neuritis attack.

### Standard protocol approvals, registrations and patient consents

The study was approved by the Ethics Committee of The First Affiliated Hospital of Fujian Medical University and written consent was obtained for all participants. Samples were deposited in a registered biobank of Fujian Institute of Neurology, the First Affiliated Hospital, Fujian Medical University.

### Statistical methods

Characteristics between patients with APO-NMOSD and NAPO-NMOSD were compared using χ2 (or Calibration χ2, Fisher exact) tests for categorical data and Student T test (or Wilcoxon rank-sum test) for continuous data. The Kaplan-Meier method was used to estimate the time to next relapsing of the subgroup without immunotherapy after first episode, and the next relapsing time of the subgroup with immunotherapy for the first-time. Predictive factors for relapsing were assessed with Cox proportional hazards regression models. In the immunotherapy subgroup, age at onset, type of initial attack, types and duration of immunotherapy were included as predictive factors for relapsing. Two-sided p values < 0.05 were considered statistically significant. Statistical analyses were performed using SPSS version 22.0.

## Results

### Demographic, clinical, and recurrent characteristics of the cohort

Clinical and demographic data of the 166 patients were summarized in Table 1. There was no difference between the two groups of gender, immunity therapy and coexisting of autoimmune disease. Compared to NAPO-NMOSD, APO-NMOSD patients were younger (34.6y VS 42.3y, *P* = 0.013), had lower EDSS at first episode (0.7 VS 4.2, *p* = 0.028) and last follow up (1.9 VS 3.3, *p* = 0.001), more likely to have multi-core characteristics at the first attack(33.3% VS 9.2%, *P* = 0.001), and have a higher annual recurrence rate (ARR) (0.4 ± 0.28 VS 0.19 ± 0.25, *P* = 0.012) (Table 1). There were 113 patients relapsed in our cohort at follow-up, APO NMOSD: NAPO-NMOSD = 21: 92. Compared with NAPO-NMOSD patients, APO-NMOSD patients had a higher proportion of brainstem involvement at first relapse (28.6% VS 4.7%, *p* < 0.001) and a lower proportion of optic nerve involvement (10.7% VS 41.1%, *p* = 0.01), with a statistically significant difference between the two groups. There was no significant difference in the ratio of spinal cord, area postrema, diencephalon/brain (Table 2).

**Table 1:** The demographics and clinical characteristics of 166 patients with AQP4-IgG (+) NMOSD

	NMOSD (*n* = 166)	APO-NMOSD (*n* = 24)	NAPO-NMOSD (*n* = 142)	*P* value
Age at onset, y, mean (SD)	41.0(13.9)	34.6(12.9)	42.3(14.1)	0.013
Female: male (ratio)	147:19(7.7:1)	20:4(5.0:1)	127:15(8.5:1)	0.602
Disease duration, m, median (range)	72(13–300)	20(14–121)	72(13–300)	<0.001
Immunity therapy ^a^, n (%)	82(49.4)	9(37.5)	73(51.4)	0.207
EDSS at first episode, mean (SD)	4.0(1.9)	0.7(0.9)	4.2(1.7)	0.028
EDSS at last follow-up, mean (SD)	3.1(2.04)	1.9(2.0)	3.3(1.97)	0.001
EDSS ≥ 6, n (%)	22(13.3)	1(4.2)	21(14.8)	0.274
VA ^b^≤10/100, n (%)	41(24.7)	3(12.5)	38(26.8)	0.214
Annualized relapse rate, mean (SD)	0.23(0.27)	0.40(0.28)	0.19(0.25)	0.012
Coexisting autoimmune diseases, n (%)	32(19.2)	6(25.0)	26(18.0)	0.442
≥ 2 core lesions ^c^ at first episode, n (%)	21(12.7)	8(33.3)	13(9.2)	0.001

Abbreviations: APO = area postrema onset; EDSS = Expanded Disability Status Scale; NAPO = non-area postrema onset; NMOSD = neuromyelitis optica spectrum disorder; VA = Visual acuity; ^a^ including azathioprine, mycophenolate mofetil and rituximab. ^b^ Only those patients who had at least 1 optic neuritis attack, visual acuity at last follow-up ≤ 10/100. ^c^ more than 2 NMOSD core syndromes in one episode.

**Table 2:** Differences between APO-NMOSD and NAPO-NMOSD patients at the site of first recurrence

	NMOSD (*n* = 135)	APO-NMOSD (*n* = 28)	NAPO-NMOSD (*n* = 107)	*P* value
Spinal cord, n (%)	68 (50.4)	11 (39.3)	57 (53.3)	0.419
Optic nerve, n (%)	47 (34.8)	3 (10.7)	44 (41.1)	0.010
Area postrema, n (%)	4 (3)	4 (14.3)	0 (0)	NA
Brainstem, n (%)	13 (9.6)	8 (28.6)	5 (4.7)	<0.001
Cerebrum/Diencephalon, n (%)	3 (2.2)	2 (7.1)	1 (0.9)	0.156

Abbreviations: APO = area postrema onset; NAPO = non-area postrema onset; NMOSD = neuromyelitis optica spectrum disorder; NA = not available

### Comparison of the risk of recurrence between APO-NMOSD and NAPO-NMOSD in the natural disease course

**Fig. 1:**
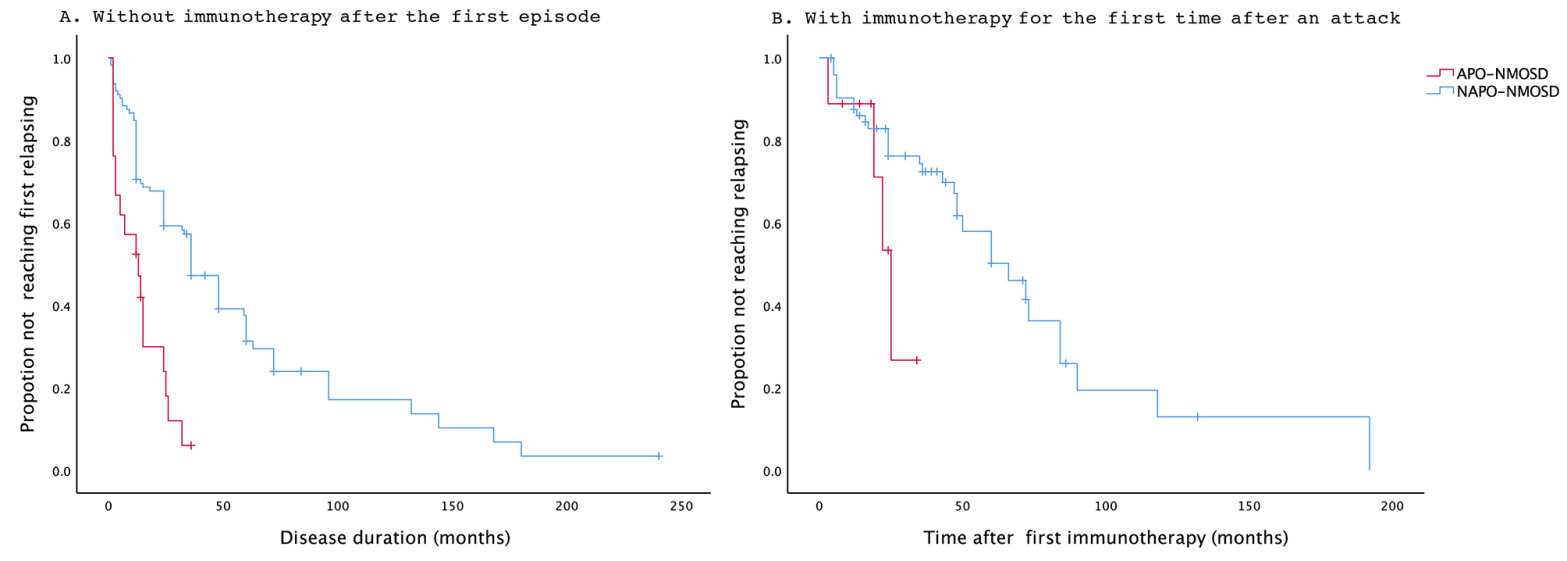
Time to next relapsing in different subgroups between APO-NMOSD and NAPO-NMOSD patients: (**A**) patients who without immunotherapy after the first episode: APO-NMOSD patients reached their next relapse earlier than NAPO-NMOSD patients (*P*<0.001). (**B**) patients who with immunotherapy for the first time after an attack: Time to next relapse was not significantly different between APO-NMOSD and NAPO-NMOSD patients (*p* = 0.073)

The differences of risk factors in different subgroups were shown in the Table 3. Compared to NAPO-NMOSD, APO-NMOSD group have a nearly threefold increased risk of having the first relapsing in patients without immunotherapy after onset in Cox proportional hazards regression models (HR 2.898, 95% CI 1.650–5.088, *p* < 0.001). Time from onset to the first relapsing in APO-NMOSD and NAPO-NMOSD patients without immunotherapy after the first episode is shown in Fig. 1A. Besides, APO-NMOSD group have a higher ARR than NAPO-NMOSD group in patients without IT during the course (0.31 ± 0.22 VS 0.16 ± 0.26, *P* = 0.038) (Table 3).

**Table 3:** The risk factors of recurrence between APO-NMOSD group and NAPO-NMOSD group in different subgroups

	NMOSD	APO-NMOSD	NAPO-NMOSD	*P* value
Patients without IT ^a^ after first episode, n	133	21	112	NA
Age at onset, y, mean (SD)	40.7(14.3)	34.2(12.6)	42.3(14.5)	0.019
Female: male (ratio)	115:18(6.4:1)	17:4(4.3:1)	98:14(7.0:1)	0.485
Patients with recurrence, n, %	96(72.2)	18(85.7)	78(69.6)	0.132
Patients without IT during the course, n	84	15	69	NA
Age at onset, y, mean (SD)	43.0(15.9)	32.9(13.1)	45.4(18.2)	0.004
Female: male (ratio)	72:12(6:1)	13:2(6.5:1)	59:10(5.9:1)	0.636
ARR, mean (SD)	0.19(0.26)	0.31(0.22)	0.16(0.26)	0.038
Patients with IT for the first-time ^b^, n	82	9	73	NA
Age at onset, y, mean (SD)	39.1(12.2)	37.4(13.0)	39.3(12.4)	0.671
RTX: IST (ratio)	15:67(1:4.5)	3:6(1:2)	12:61(1:5.1)	0.355
IT start time, m, median (range)	1-264(13)	1–71(3)	1-264(20)	0.825
Patients with recurrence, n, %	37(45.1)	4(44.4)	33(45.2)	0.965

Abbreviations: APO = area postrema onset; NAPO = non-area postrema onset; NMOSD = neuromyelitis optica spectrum disorder; RTX = rituximab; IT: immunotherapy; IST = immunosuppressive therapy; ARR = Annual recurrence rate; NA = not available. ^a^ including rituximab and immunosuppressive therapy azathioprine and mycophenolate mofetil; ^b^ only records the type of first immunotherapy, the time to initiate treatment after onset, and the proportion of recurrence after first immunotherapy.

### Comparison of the risk of recurrence between APO-NMOSD and NAPO-NMOSD patients with immunotherapy for the first-time

The risk to the next relapsing was increased by 2.5-fold for APO-NMOSD patients with immunotherapy compared with NAPO-NMOSD patients, but the difference was not statistically significant (HR 2.5, 95% CI 0.801–7.712, *P* = 0.115). Kaplan-Meier showed no significant difference in the next recurrence time between the two groups with immunotherapy (*P* = 0.073) (Fig. 1B).

### The demographic and recurrent characteristics of APO-NMOSD, ONO-NMOSD and MO-NMOSD patients

**Fig. 2:**
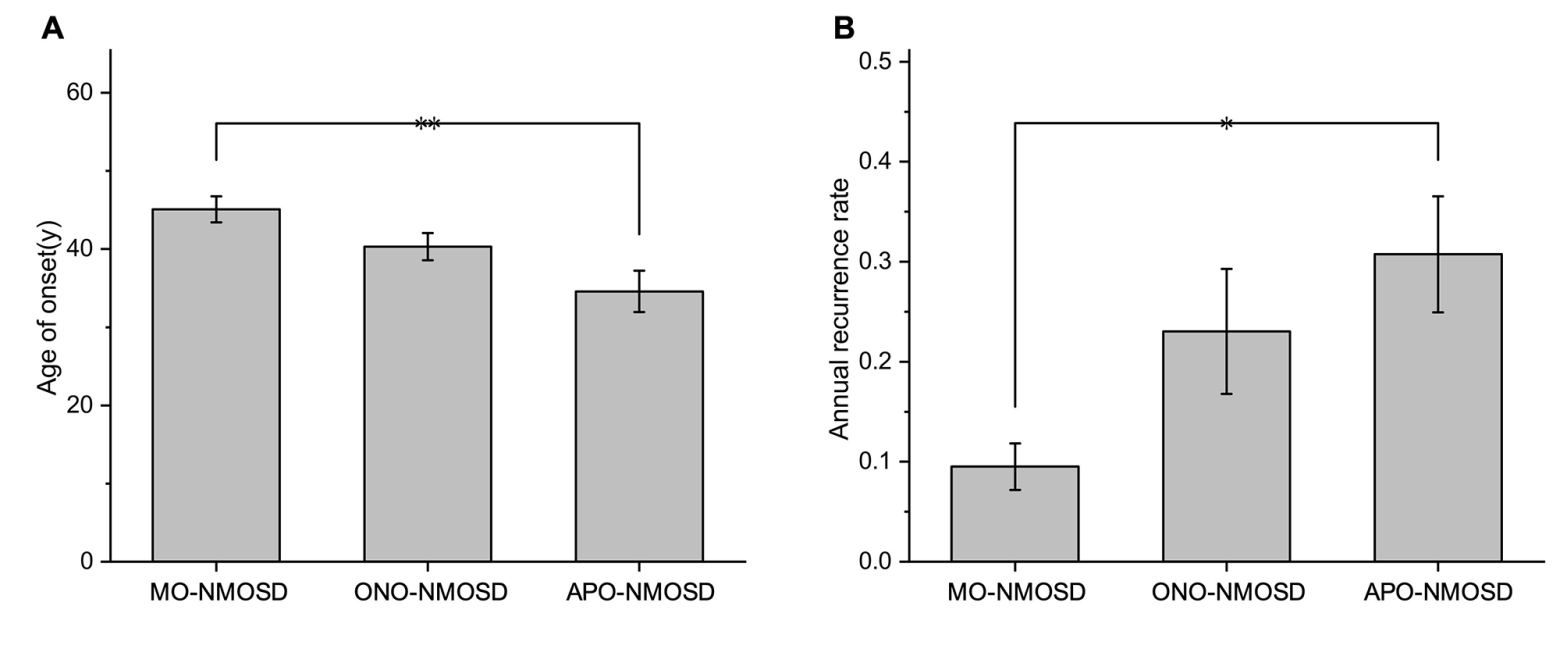
Age of onset and annual recurrence rate in APO-NMOSD, ONO-NMOSD and MO-NMOSD groups. *p < 0.05; **p < 0.01; ***p < 0.001. (**A**) shows the age of onset of the APO-NMOSD, ONO-NMOSD and MO-NMOSD groups. (**B**) shows the annual recurrence rate of patients without immunotherapy in the APO-NMOSD, ONO-NMOSD and MO-NMOSD groups

**Fig. 3:**
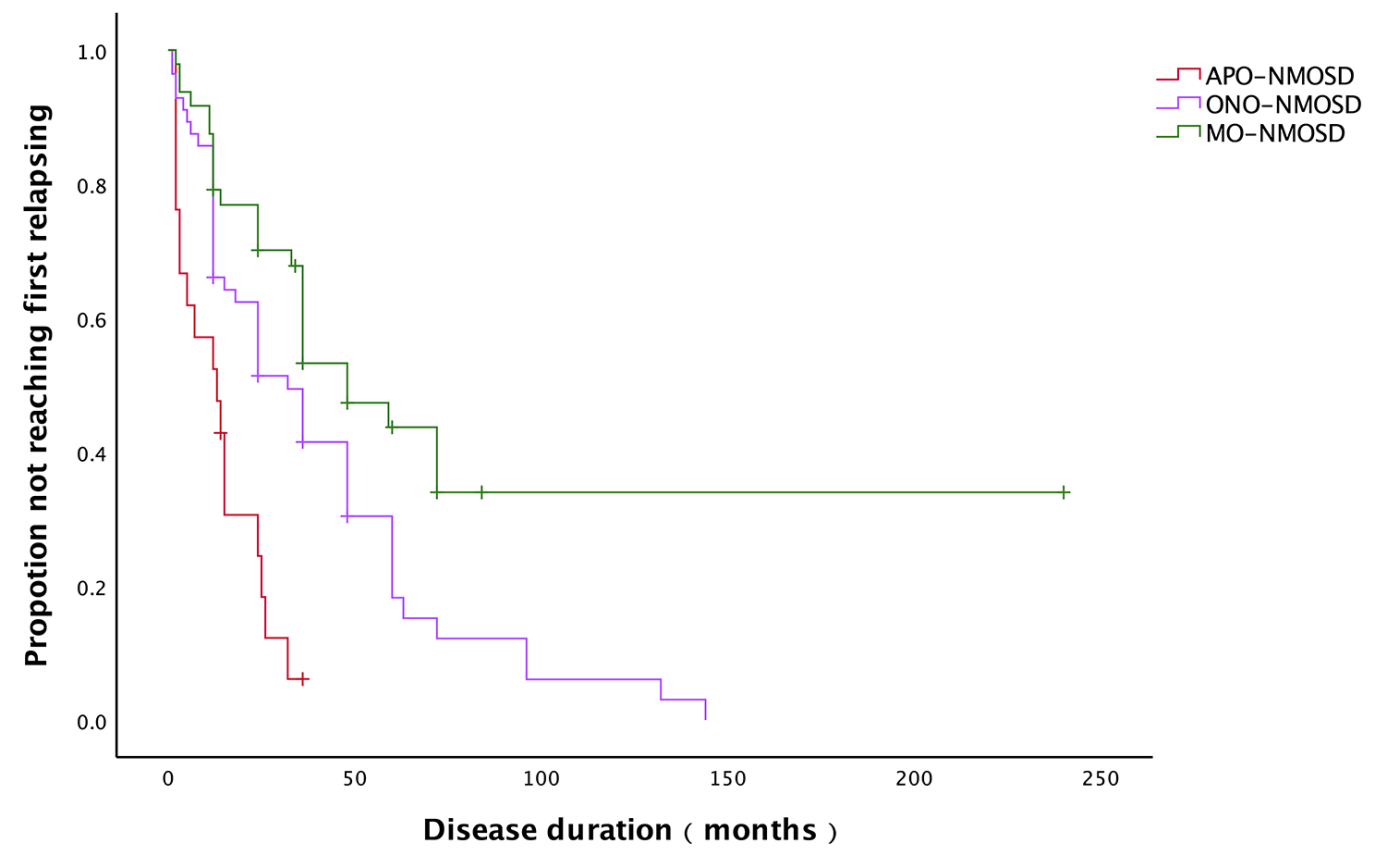
Time to first relapsing in patients without immunotherapy after the first episode between APO-NMOSD, ONO-NMOSD and MO-NMOSD groups. APO-NMOSD patients reached the first relapsing earlier than ONO-NMOSD and MO-NMOSD patients (*P*<0.001)

Compared to APO-NMOSD, myelitis onset (MO)-NMOSD patients were older (45.1y VS 34.6y, *P*<0.01) and have a lower ARR in patients without IT during the course (0.10 ± 0.13 VS 0.31 ± 0.22, *P*<0.05). Optic neuritis onset (ONO)-NMOSD group was also older (40.3y VS 34.6y, *P*>0.05) and have a lower ARR (0.23 ± 0.34 VS 0.3 ± 0.22, *P*>0.05), but the differences were not statistically significant. There was no difference between the three groups of gender. (Table 4; Fig. 2 provide additional details on these findings). Patients with APO-NMOSD have higher risk of having the first relapsing compared to optic neuritis onset-NMOSD (HR 2.641, 95% CI 1.427–4.887, *p* = 0.002) and myelitis onset-NMOSD patients (HR 3.593, 95% CI 1.736–7.438, *p* = 0.001) without immunotherapy after onset. Time from onset to the first relapsing of the three groups are shown in Fig. 3.

**Table 4:** The demographic and recurrent characteristics of APO-NMOSD, ONO-NMOSD and MO-NMOSD groups

	APO-NMOSD (*n* = 24)	ONO-NMOSD (*n* = 65)	MO-NMOSD (*n* = 67)	*P* value
Age at onset, y, mean (SD)	34.6(12.9)	40.3(14.1)	45.1(13.6)	0.005
Female: male (ratio)	20:4(5.0:1)	57:8 (7.1:1)	62:5(12.4:1)	0.182
Patients without IT during the course, n	15	30	34	NA
ARR, mean (SD)	0.31(0.22)	0.23(0.34)	0.10(0.13)	<0.001
Patients without IT after first episode, n	21	56	48	NA
Patients with recurrence, n, %	18(85.7)	46(82.1)	26(54.2)	0.002

Abbreviations: APO = area postrema onset; ONO = optic neuritis onset; MO = myelitis onset; NMOSD = neuromyelitis optica spectrum disorder; IT = immunotherapy; ARR = annual recurrence rate; NA = not available.

## Discussion

The first attack of AQP4-IgG seropositive NMOSD are most commonly optic neuritis (45%) and myelitis (47%), followed by APS (9.4-14%) [6, 14], only a small number of patients presenting with isolated brainstem, brain or diencephalon damage [14, 15]. The onset of APS is a very important clinical type of the disease spectrum. Previous studies have shown that the main pathological features of AP in AQP4-IgG seropositive NMOSD patients are inflammation and demyelination rather than necrosis [16, 17], this may explain the complete recovery of refractory hiccup, vomiting and nausea after treatment and the reversibility of the lesion in APS patients. Because of non-specific symptoms of neurological deficits, APO-NMOSD often leads to delayed diagnosis and treatment, resulting in disease progression [2, 4], as our study found that APO-NMOSD patients have lower treatment rates and higher multi-lesion involved in the acute phase. Early identification and intervention are important for preventing disease progression.

The first recurrence of APO-NMOSD patients is characterized by spatial and temporal adjacency in our study, with shorter relapsing time and adjacent areas like brainstem, this may be related to the.

direct spread of AP inflammation because of the particularity of AP anatomy mentioned above [5, 6].

The mean age of onset in APO-NMOSD was younger than NAPO-NMOSD in our study, it suggested that AP lesions may appear in early course of the disease, or be more likely to involve younger patients, so it is valuable to give them adequate attention and timely intervention. Although the first episode of AP patients may have lower disability, but their high annual recurrence rate is an important factor affecting the prognosis, therefore, early immunosuppressive therapy is necessary to reduce the risk of relapse and to avoid the accumulation of relapse-related disability.

Studies have demonstrated that race, sex, age, attack types and immunosuppressive therapy were associated with the prognosis of NMOSD [18–21]. Optic neuritis and myelitis are major factors for poor prognosis and poor quality of life [18, 22]. Medullary lesions had higher annual recurrence rates and higher EDSS scores [23, 24], while some researchers found that patients with brainstem lesions had lower EDSS scores and a lower risk of having an EDSS > 6 score [25]. In our study, APO-NMOSD patients seem to have better disability outcomes during limited follow-up time, this may be one of the reasons for a good prognosis that younger APO patients have a better capacity to compensate for damage after attack [26]. In addition, APS injury is mild and can be fully recovered, while the NAPO-NMOSD patients is more likely to severe disability caused by optic neuritis and myelitis in subsequent episodes. The course of the disease is a main factor affecting the prognosis of disability, so longer observation is still needed to clarify.

## Conclusions

AQP4-IgG seropositive APO-NMOSD patients are a younger group with a higher risk of recurrence. Clinicians should be more vigilant about AP damage in NMOSD for its potential hazards. This study was based on a single-center sample, but we hope that it will shed light on the disease surveillance, treatment strategy development of NMOSD.

## Data Availability

All data generated or analysed during this study are included in this published article.

## Abbreviations

NMOSD: Neuromyelitis optica spectrum disorder
AQP4: Aquaporin 4
AP: Area postrema
APO: Area postrema onset
NAPO: Non-area postrema onset
ONO: Optic neuritis onset
MO: Myelitis onset
EDSS: Expanded Disability Status Scale
VA: Visual acuity
RTX: Rituximab
IST: Immunosuppressive therapy
IT: Immunotherapy
ARR: Annual recurrence rate
